# Estimating immunity with mathematical models for SARS-CoV-2 after COVID-19 vaccination

**DOI:** 10.1038/s41541-023-00626-w

**Published:** 2023-03-06

**Authors:** Yoshifumi Uwamino, Kengo Nagashima, Ayumi Yoshifuji, Shigeru Suga, Mizuho Nagao, Takao Fujisawa, Munekazu Ryuzaki, Yoshiaki Takemoto, Ho Namkoong, Masatoshi Wakui, Hiromichi Matsushita, Naoki Hasegawa, Yasunori Sato, Mitsuru Murata

**Affiliations:** 1 Department of Laboratory Medicine, Keio University School of Medicine, 35 Shinanomachi, Shinjuku-ku, Tokyo, Japan; 2 Department of Infectious Diseases, Keio University School of Medicine, 35 Shinanomachi, Shinjuku-ku, Tokyo, Japan; 3 Biostatistics Unit, Clinical and Translational Research Center, Keio University Hospital, 35 Shinanomachi, Shinjuku-ku, Tokyo, Japan; 4 Department of Nephrology, Tokyo Saiseikai Central Hospital, 1-4-17 Mita, Minato-ku, Tokyo, Japan; 5 Infection Control Committee, Japanese Society for Dialysis Therapy, 2-38-21 Hongo, Bunkyo-ku, Tokyo, Japan; 6 National Hospital Organization Mie National Hospital, 357 Osatokubotacho, Tsu, Mie Japan; 7 Department of Preventive Medicine and Public Health, Keio University School of Medicine, 35 Shinanomachi, Shinjuku-ku, Tokyo, Japan; 8 Clinical Research Center for Medicine, International University of Health and Welfare, 4-1-26 Akasaka, Minato-ku, Tokyo, Japan

**Keywords:** Outcomes research, Experimental models of disease

## Abstract

Tools that can be used to estimate antibody waning following COVID-19 vaccinations can facilitate an understanding of the current immune status of the population. In this study, a two-compartment-based mathematical model is formulated to describe the dynamics of the anti-SARS-CoV-2 antibody in healthy adults using serially measured waning antibody concentration data obtained in a prospective cohort study of 673 healthcare providers vaccinated with two doses of BNT162b2 vaccine. The datasets of 165 healthcare providers and 292 elderly patients with or without hemodialysis were used for external validation. Internal validation of the model demonstrated 97.0% accuracy, and external validation of the datasets of healthcare workers, hemodialysis patients, and nondialysis patients demonstrated 98.2%, 83.3%, and 83.8% accuracy, respectively. The internal and external validations demonstrated that this model also fits the data of various populations with or without underlying illnesses. Furthermore, using this model, we developed a smart device application that can rapidly calculate the timing of negative seroconversion.

## Introduction

Two mRNA vaccines for SARS-CoV-2, BNT162b2 (Pfizer-BioNTech) and mRNA-1273 (Moderna), have demonstrated significant effectiveness after only two doses of vaccine^1,2^. Since the waning of immunogenicity was reported during the time course^3,4^, the administration of booster doses was accelerated worldwide. For example, the Center for Disease Control and Prevention (USA) recommended that people who have received two doses of BNT162b2 receive a third dose at least 5 months after receiving the second dose^5^. The recommended timing of booster dose administration was determined primarily based on the results of studies assessing vaccine effectiveness during the time course^6–8^. However, conducting population-based vaccine effectiveness studies might be practically difficult in certain countries because the researchers must track the occurrences of infection within a large population of vaccinated people for a long time. Antibody concentrations (titers) and antispike protein immunoglobulin G (IgG) are related to protection against the infection^9,10^; therefore, measuring the concentration of antibodies in vaccinated people would be helpful in developing public health policies about vaccination and infection control. However, repetitive testing is expensive. Therefore, models for estimating the future dynamics of antibodies are required. In addition, it is difficult for the general population to perceive the waning of immunity following vaccination, which might be one of the reasons for unwillingness to take the booster dose^11^. The development of digital tools for estimating individual antibody dynamics might lead to a better understanding of waning immunity following vaccination, implying the need for an increased rate of booster vaccination, which remains low in several countries^12^.

In this study, using the vaccinated cohort data, we develop and validate a mathematical model for estimating antibody waning following vaccination. Hence, we establish a prototype smart phone application to estimate the future waning of antibodies based on a single measurement of a SARS-CoV-2 antibody titer.

## Results

### Existing Data

**Fig. 1:**
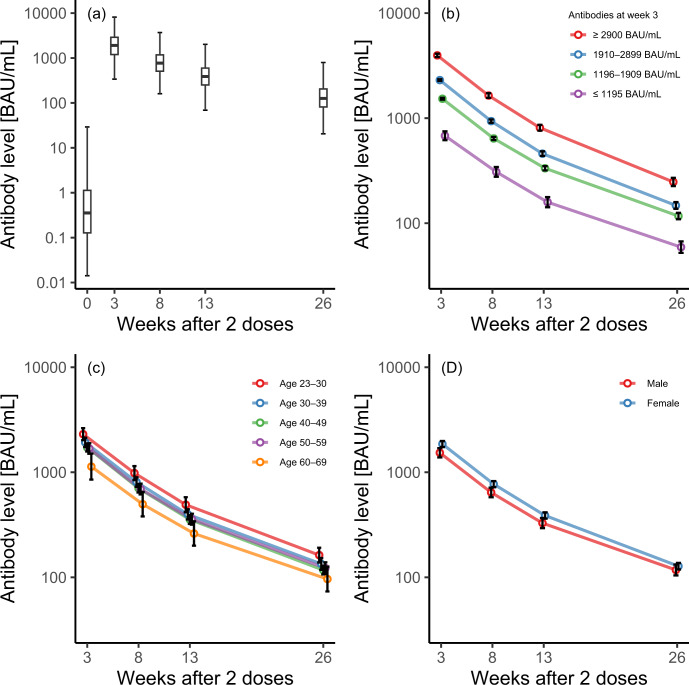
Semi-logarithmic plots of SARS-CoV-2 spike antibodies after two doses of BNT162b2 vaccine. **a** Box plots of all available cases (*N* = 657; cases for which data were available only at week zero were excluded); logarithmic means stratified by **b** antibodies at week 3, **c** age group, and **d** sex. Error bars indicate 95% confidence intervals. **b** The cutoffs for antibodies at week 3 were determined based on the 25th, 50th, and 75th percentiles. All the demonstrated antibody titers were anti-receptor-binding domain IgG.

Figure 1 depicts semi-logarithmic plots of antibody titers for healthy medical workers at Keio University Hospital (*N* = 657) after two doses of the BNT162b2 vaccine. Figure 1a indicates that the antibody titer increased after two doses and subsequently decreased from 3 to 26 weeks. The rate of decrease was similar from 3 to 13 weeks and slightly lower after 13 weeks. Figure 1b–d indicate the average antibody titer stratified by groups defined by antibody titer at week 3, age, and sex. The characteristics differed for each group, but the rates of decrease were not different. The differences in the antibody titers were the largest when the data were grouped by antibody titer at week 3.

### Model Selection

We constructed three candidate models with the objective of developing a mathematical model to describe and predict individual antibody titers following two doses: (1) one-compartment model, (2) two-compartment model, and (3) double exponential model. We selected (1) and (2) as the candidate models to represent the elimination of antibodies generated in the body as they transition between compartments. We selected (3) as a nonlinear model to describe the change in the antibody titer (that is, the responses) over time. Further details on the model structures and hyperparameters are provided in the Methods section. We fitted the three candidate models to data obtained from participants at Keio University Hospital (*N* = 657) and found that model (2) demonstrated the smallest leave-one-out cross-validation information criterion (LOOIC) and best-fit (see Table 1). This result was consistent with the trend of the rate of decrease, as depicted in Fig. 1 (a constant rate of decrease from 3 to 13 weeks, with a slightly lower rate after 13 weeks). For the best-fit model, the convergence of the Markov chain Monte Carlo samplers was achieved and sufficient quality was demonstrated (see Supplementary Figs. 1–4 and Supplementary Table 2).

**Table 1:** Results of model comparison.

	LOOIC
(1) One-compartment model	44350.3
(2) Two-compartment model	**38810.6**
(3) Double exponential model	43491.4

LOOIC (leave-one-out cross-validation information criterion) is a measure of the goodness of fit of a model, with smaller values indicating a better fit. The two-compartment model LOOIC was the smallest and emphasized, therefore, the best fit.

Bold means the best fit with the minimum value.

### Model validation

We applied a further test of the prediction performance of the two-compartment model through 10-fold cross-validation on the dataset from the participants at Keio University Hospital and obtained an accuracy of 97.0% (95% confidence interval (CI): 95.2%–98.1%), RMSE of 0.430, and Pearson’s correlation coefficient of 0.841 (Table 2). We defined accuracy as follows: accuracy = ((no. of patients whose 95% prediction interval included the actual value)/(no. patients)) × 100 [%].

**Table 2:** Results of internal and external validations.

	Data	Accuracy (95% CI)	RMSE	Pearson’s correlation coefficient
Internal validation	Cross-validation: healthy medical workers (*N* = 657)	97.0% (95.2%, 98.1%)	0.430	0.841
External validation	Mie National Hospital: healthy medical workers (input data for 3.5 weeks; *N* = 165)	98.2% (94.4%, 99.5%)	0.508	0.709
External validation	Mie National Hospital: healthy medical workers (input data for 12 weeks; *N* = 165)	94.5% (89.9%, 97.5%)	0.484	0.819
External validation	JSDT: hemodialysis patients (*N* = 192)	83.3% (77.1%, 88.2%)	0.924	0.721
External validation	JSDT: nondialysis elderly patients (*N* = 100)	83.8% (75.0%, 90.3%)	0.979	0.553

Accuracy = ((no. of patients whose 95% prediction interval included the actual value)/(no. of patients)) × 100 [%], and ${{{\mathrm{RMSE}}}}_t = \sqrt {{\textstyle{1 \over n}}\mathop {\sum}\nolimits_{i = 1}^n {\left[ {\log Y_{it} - \log \left\{ {{{{\mathrm{median}}}}\left( {\hat Y_{it}} \right)} \right\}} \right]^2} }$. The RMSE and Pearson’s correlation coefficient between log *Y*_*it*_ and $\log \left\{ {{{{\mathrm{median}}}}\left( {\hat Y_{it}} \right)} \right\}$ at 26 weeks were indicated for cross-validation, and RMSE and Pearson’s correlation coefficient at 24 weeks were indicated for Mie National Hospital and JSDT.

*JSDT* The Japanese Society for Dialysis Therapy.

Furthermore, we verified the prediction performance of the model using external data. We found that, at 24 weeks, the model demonstrated a prediction accuracy of 98.2% (95% CI: 94.4%–99.5%), RMSE of 0.508, and Pearson’s correlation coefficient of 0.709 for healthcare workers from the Mie National Hospital (*N* = 165) with 3.5 weeks of input data; prediction accuracy of 94.5% (95% CI: 89.9%–97.5%), RMSE of 0.484, and Pearson’s correlation coefficient of 0.819 for healthcare workers from the Mie National Hospital (*N* = 165) with 12 weeks of input data; prediction accuracy of 83.3% (95% CI: 77.1%–88.2%), RMSE of 0.924, and Pearson’s correlation coefficient of 0.721 for dialysis patients from the Infection Control Committee of the Japanese Society for Dialysis Therapy (JSDT; *N* = 192); and prediction accuracy of 83.3% (95% CI: 75.0%–90.3%), RMSE of 0.979, and Pearson’s correlation coefficient of 0.553 for nondialysis elderly patients from JSDT (*N* = 100) (Table 2). For the participants in the JSDT cohort whose predictions failed, most of the predicted values were higher than the actual values.

### Prediction results

**Fig. 2:**
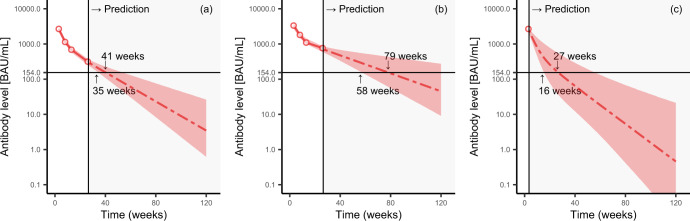
Prediction results. Prediction results from fitting the best-fit model to three selected participants from Keio University Hospital. The area to the right of the vertical reference line displays the predicted results. Data and predicted results of antibody titers (anti-receptor-binding domain IgG) for a participant whose antibody titer increased to approximately 2500 BAU/mL (**a**) and 3500 BAU/mL (**b**) after two vaccination doses. **c** Data and predicted results for a participant with only one measurement available (week 3) owing to missing data. Time instants are shown when the lower limit of the 95% prediction interval and the median value falls below the 154 BAU/mL threshold, the horizontal reference line, for classification as a protective antibody titer. The vertical axis represents the logarithmic scale.

Figure 2 presents the prediction results from fitting the two-compartment model to three selected participants from Keio University Hospital. We randomly selected participants whose antibody titer increased to ~2500 BAU/mL (Fig. 2a, c) and 3500 BAU/mL (Fig. 2b) after two vaccinations. The predicted results for the participants with higher antibody titers at 3 weeks suggested that they maintained higher antibody titers (Fig. 2a, b). The two-compartment model fitted well, as the change in the rate of decrease in the antibody titers from 3 and 13 weeks and after 13 weeks was accurately modeled (Fig. 2a, b).

Because we used a hierarchical Bayes model, we could obtain prediction results for the participants with only one measurement point owing to missing data or a new participant (see Fig. 2c and Supplementary Figs. 5–7). The time instants are indicated when the lower limit of the 95% prediction interval and the median value fall below the 154 BAU/mL threshold for classification as a protective antibody titer (Fig. 2a–c). The subjects with higher antibody titers in the third week crossed the classification threshold later than those with lower antibody titers at the third week. As the participant indicated in Fig. 2c had only one point of data, the width of the prediction interval was wide, reflecting the small amount of information available.

**Fig. 3:**
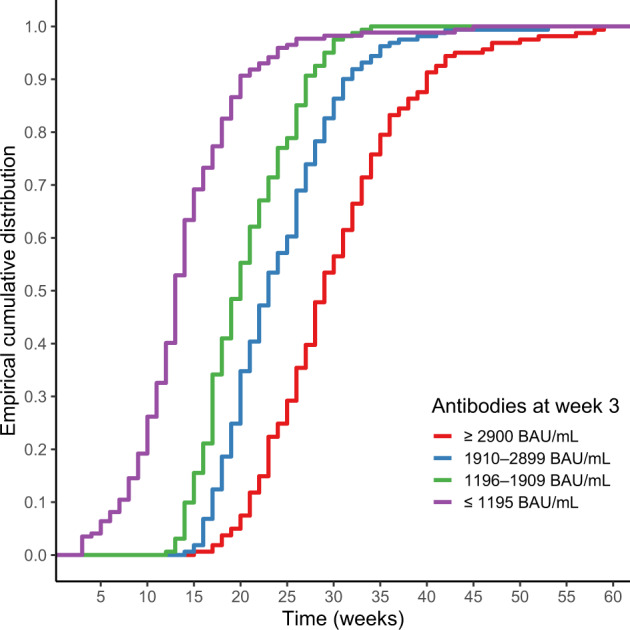
Empirical distribution functions of predicted results of time instants at which antibody titers fall below the positive threshold. Empirical distribution functions of the lower limit of the 95% prediction interval for each participant falling below the 154 BAU/mL threshold for classification as a protective antibody titer, stratified by antibodies (anti-receptor-binding domain IgG) at week 3 (*N* = 657). The cutoffs for antibodies at week 3 were determined based on the 25th, 50th, and 75th percentiles.

Figure 3 depicts the distribution of the time points at which the lower limit of the 95% prediction interval for each participant fell below the protective classification threshold. Participants with high antibody titers at week 3 had a delayed fall below the protective threshold. This result was consistent with the trend in the group differences indicated in Fig. 1b. Comparing the groups with the lowest and highest antibody titers at week 3, we see that the distribution peak differs by ~25 weeks.

### Development of smart device application

**Fig. 4:**
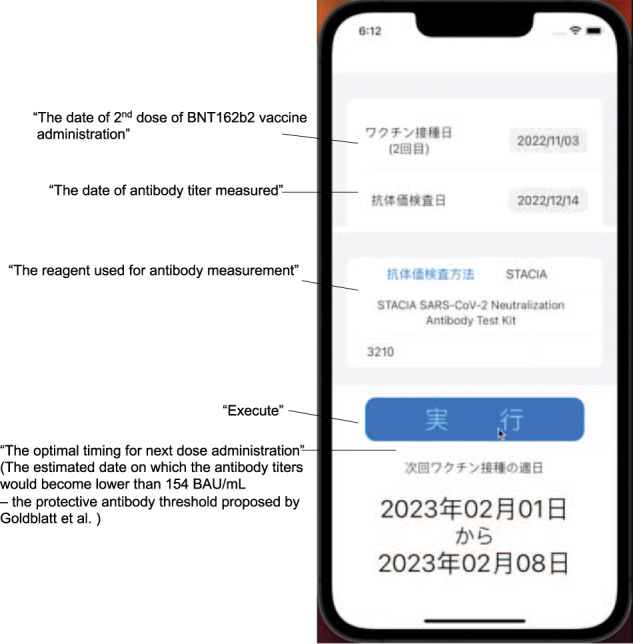
Prototype of antibody simulation application using model. Screen image of the iOS application named “COVID Vaccine Navi.” Information about the date of vaccination singly measured antibody titer, and estimated date of negative seroconversion is presented. A demonstration video is available in the supplementary material.

The prototype of an iOS-based smart device application was built using the proposed model. The user was required to input the date of their second BNT162b2 vaccine dose, the date of their antibody titer measurement, the type of reagent used for the antibody titer measurement, and the antibody titer. Subsequently, the antibody dynamic was simulated by the model built into the application, and the estimated date on which the antibody titers would become lower than 154 BAU/mL, which was the protective antibody threshold proposed by Goldblatt et al.^13^, was displayed (Fig. 4 and Supplementary video). The protective antibody threshold was still to be fixed; it could be variable based on the epidemic variants. Therefore, the administrator could freely adjust the threshold based on advice from public health authorities.

## Discussion

Although mathematical models are commonly used for estimating drug concentration in pharmacodynamics, their application in estimating antibody dynamics following vaccination is rare. Our model successfully simulated the individual waning curves of antibody titers following the administration of mRNA COVID-19 vaccine, which was verified by various datasets. We applied the pharmacodynamic model to understand the dynamics of antibody-titer-induced humoral immunity. This model was originally designed to estimate the concentration of an administered compound or its metabolites. Therefore, it is interesting that it can be used for estimating the antibody titer produced by vaccination, which is neither the administered compound (the vaccine itself) nor its metabolites (RNA). In the generation of antibodies following vaccination, far more complicated processes such as transcription, antigen presentation, and antigen-specific immune cell inductions are included than in the kinetics of other medications, such as antibiotics.

Regarding the application of pharmacodynamic models to antibody dynamics, Favresse et al.^14^ discussed the suitability of the one-compartment model for antibody waning over 3 months after BNT162b2 vaccination. However, the present study demonstrated better predictability using the two-compartment model as compared with the one-compartment one.

Two-compartment models are often used as pharmacodynamic models that have two different distribution areas, such as plasma and target organs, for medication with various metabolizations. Although the reason that the two-compartment model demonstrated better predictability is unclear, we hypothesized that two different types of IgG production kinetics mimic two different distribution areas, which we named “two immunological compartments.” The first “compartment” refers to IgG production by immature memory B cells stimulated by the second dose of the vaccine. Immature memory B cells specific to SARS-CoV-2 spike proteins are differentiated after the first dose of the vaccine. Following the second dose, the SARS-CoV-2 spike-protein-specific immature memory B cells rapidly produce IgG for SARS-CoV-2 spike proteins with low avidity. These immature memory B cells are not long-lasting; therefore, the antibody titer in this compartment declines rapidly. The second “compartment” is IgG production by plasma cells. Some of the immature memory B cells specific to SARS-CoV-2 spike proteins are selected and matured in the germinal center through the stimulation of antigen-peptide-translated mRNA from the second dose and differentiated into long-term memory and plasma cells. The plasma cells can continuously produce high-avidity IgG specific to SARS-CoV-2 spike proteins without antigen stimulation. The IgG produced by the plasma cells is long-lasting; therefore, the antibody titer in this compartment declines slowly. Although further investigation is essential for validating this hypothesis, the “two immunological compartments” approach, consisting of memory B cells and long-lived plasma cells, might contribute to research on the suitability of two-compartment models^15–17^. Although simpler antibody kinetics were demonstrated through the mathematical modeling study of antibody kinetics following vaccinia virus vaccine administration when compared with the proposed model^18^, it is difficult to compare humoral immunity generated by mRNA-based SARS-CoV-2 vaccines and live-attenuated vaccinia virus vaccines, which are considered to induce life-long immunity. In addition, the observation periods in our study were much longer than in the study of the vaccinia virus.

The data obtained in this study suggest that antibody titers differ more considerably between individuals rather than based on factors such as age and gender. We confirmed through internal and external validations that a hierarchical Bayesian model can account for individual differences and be used for predictions with a high accuracy rate. The hierarchical Bayesian model can also predict changes in future antibody titers based on a single point of measurement data.

The datasets used for building the models and for external validation provided by Mie National Hospital consisted mostly of data from healthy young or middle-aged people. However, the JSDT cohort consisted of 192 elderly dialysis patients receiving hemodialysis (HD) and 100 elderly patients with some underlying diseases such as diabetes and hypertension. Although insufficient antibody production was reported among HD, diabetic, and hypertensive patients^19–22^, our external validation demonstrated as high as 80% accuracy, suggesting that the proposed model is effective, even in groups with underlying diseases.

Our study had three limitations: First, all the participants in the study were administered the BNT162b2 vaccine. Therefore, it is uncertain whether our model can be used for people who were administered other types of COVID-19 vaccines and were younger than the participants in the selected cohorts, that is, children and juveniles. Second, the proposed model could only be validated for up to 26 weeks based on the available data. Therefore, it is unclear how accurate the estimates will be in later time instances. Finally, the model cannot estimate the antibody titer after the administration of the booster dose. As a large number of people are being administered booster doses worldwide, the modification and validation of the proposed model using a dataset of antibodies from people who have received various types of COVID-19 vaccines and those who have received booster doses are warranted.

In conclusion, the anti-SARS-CoV-2 antibody dynamics of healthy adults vaccinated with two doses of BNT162b2 vaccine were described accurately using a mathematical model based on the two-compartment model.

## Methods

### Antibody-titer datasets for model construction

To construct the mathematical models, we obtained consecutively measured antibody titers from a prospective cohort study of BNT162b2-vaccinated healthcare providers. The study included 673 participants who had received two doses of BNT162b2 vaccine at Keio University Hospital (Tokyo, Japan) (Supplementary Table 1). Five serum samples were collected from each participant before vaccination and then 3 weeks, 8 weeks, 3 months, and 6 mo after the administration of the two doses. The IgG antibody titers for the receptor-binding domain of the SARS-CoV-2 spike proteins were measured using Alinity SARS-CoV-2 IgG II reagents and an Alinity analyzer (Abbott; IL, USA). The study protocol was approved by the Ethics Committee of Keio University School of Medicine (approval No. 20210301), and written informed consent was obtained from all the participants. The measured antibody titers in the original AU/mL units were converted into BAU/mL using the conversion formula 1 BAU/mL = 0.142 AU/mL, following the manufacturer’s instructions.

### Antibody datasets for external validation

To externally validate the model, antibody titer data were obtained from 165 healthy healthcare providers who had received two doses of BNT162b2 vaccine at the National Health Organization Mie National Hospital (Mie, Japan). The IgG antibody titer for SARS-CoV-2 spike proteins of consecutively obtained serum samples was measured using an enzyme-linked immunoassay-based kit (Denka Co. Ltd, Tokyo, Japan), which has been certified by WHO international standard serum samples. Four serum samples were collected from each subject before vaccination and then 3.5 weeks, 3 months, and six 6 months after the administration of the two doses. The study protocol was approved by the Ethics Committee of the National Hospital Organization Mie National Hospital (Approval No. 2021-141), and written informed consent was obtained from all the participants.

In addition, the antibody titers of nondialysis elderly patients (*N* = 100) and HD patients (*N* = 192) obtained from the JSDT were measured using the Ortho-Clinical Diagnostics VITROS® Anti-SARS-CoV-2 IgG Chemiluminescent Immunoassay, which has been certified by WHO international standard serum samples. Four serum samples were collected from each subject before vaccination and then 2 weeks, 3 months, and 6 months after the administration of the two doses. This study was approved by the Ethics Committee of the JSDT (Approval Nos 1–10), and written informed consent was obtained from all the participants (Supplementary Table 1).

### Model development

To describe and predict individual antibody titers after two doses of BNT162b2 vaccine, we constructed three hierarchical Bayes models: (1) one-compartment model, (2) two-compartment model, and (3) double exponential model. A compartmental model, such as (1) and (2), is a type of differential equation model used to describe how materials transition among the compartments of a system. To represent the elimination of antibodies, we selected (1) and (2) as candidate models of the antibodies transitioning among compartments. Model (3) is a Weibull-type model used to model dose and time responses, and it was selected as a nonlinear model to describe the change in antibody titers (that is, response) over time. We also considered other models, including other Weibull-type models, and age and/or sex as covariates, but these models did not converge as well. We subsequently determined the best-fit model among the three candidate models and evaluated the internal and external validities of the best model.

Let *Y*_*it*_ denote the observation vector for the *i*th subject (*i* = 1, 2, …, *n*) at time *t*, where *t* is the number of weeks following the second vaccine dose (*t* = 3, 8, 13, 26). We used the following hierarchical Bayes models:

$$\log Y_{it} \sim {{{\mathcal{N}}}}\left( {\log f_{\left( j \right)}\left( {t\left| {{{{\mathbf{\uptheta }}}}_{ij}} \right.} \right),\sigma _Y^2} \right),$$

$$\theta _{ij} \sim {{{\mathcal{N}}}}\left( {{{{\mathbf{\upmu }}}}_j,{{{\mathbf{{\Sigma}}}}}_j} \right),$$

where *f*_(*j*)_ denotes a nonlinear regression function and **θ**_*ij*_ represents the parameter vector of the *i*th subject, which specifies the nonlinear regression function. We define *f*_(*j*)_ and **θ**_*ij*_ for each model as follows:

1. One-compartment model:
  $$f_{\left( 1 \right)}\left( {t\left| {{{{\mathbf{\uptheta }}}}_{i1}} \right.} \right) = \exp \left( {a_{i1}} \right)\exp \left( { - b_{i1}t} \right),$$
  $${{{\mathbf{\uptheta }}}}_{i1} = \left( {a_{i1},b_{i1}} \right),$$
  $${{{\mathbf{\upmu }}}}_1 = \left( {\mu _a,\mu _b} \right)^T,$$
  $${{{\mathbf{{\Sigma}}}}}_1 = \left( {\begin{array}{*{20}{c}} {\sigma _a^2} & {\rho \sigma _a\sigma _b} \\ {\rho \sigma _a\sigma _b} & {\sigma _b^2} \end{array}} \right);$$
2. Two-compartment model:
  $$f_{\left( 2 \right)}\left( {t\left| {{{{\mathbf{\uptheta }}}}_{i2}} \right.} \right) = \exp \left( {a_{i2}} \right)\exp \left( { - b_{i2}t} \right) + \exp \left( {c_{i2}} \right)\exp \left( { - d_{i2}t} \right),$$
  $${{{\mathbf{\uptheta }}}}_{i2} = \left( {a_{i2},b_{i2},c_{i2},d_{i2}} \right),$$
  $${{{\mathbf{\upmu }}}}_2 = \left( {\mu _a,\mu _b,\mu _c,\mu _d} \right)^T,$$
  $${{{\mathbf{{\Sigma}}}}}_2 = \left( {\begin{array}{*{20}{c}} {{{{\mathbf{{\Sigma}}}}}_{21}} & {{{\mathbf{0}}}} \\ {{{\mathbf{0}}}} & {{{{\mathbf{{\Sigma}}}}}_{22}} \end{array}} \right) = \left( {\begin{array}{*{20}{c}} {\sigma _a^2} & {\rho _1\sigma _a\sigma _b} & 0 & 0 \\ {\rho _1\sigma _a\sigma _b} & {\sigma _b^2} & 0 & 0 \\ 0 & 0 & {\sigma _c^2} & {\rho _2\sigma _c\sigma _d} \\ 0 & 0 & {\rho _2\sigma _c\sigma _d} & {\sigma _d^2} \end{array}} \right);$$
3. Double exponential model:
  $$f_{\left( 3 \right)}\left( {t\left| {{{{\mathbf{\uptheta }}}}_{i3}} \right.} \right) = \exp \left\{ {\exp \left( {a_{i3}} \right)\exp \left( { - b_{i3}t} \right)} \right\} - 1,$$
  $${{{\mathbf{\uptheta }}}}_{i3} = \left( {a_{i3},b_{i3}} \right),$$
  $${{{\mathbf{\upmu }}}}_3 = \left( {\mu _a,\mu _b} \right)^T,$$
  $${{{\mathbf{{\Sigma}}}}}_3 = \left( {\begin{array}{*{20}{c}} {\sigma _a^2} & {\rho \sigma _a\sigma _b} \\ {\rho \sigma _a\sigma _b} & {\sigma _b^2} \end{array}} \right);$$

We used weak informative priors as follows: $\mu _a,\mu _b,\mu _c,\mu _d \sim {{{\mathcal{N}}}}\left( {0,100^2} \right)$, $\sigma _a,\sigma _b,\sigma _c,\sigma _d \sim {{{\mathrm{HalfCauchy}}}}\left( {0,50} \right)$, and ${{{\mathbf{{\Sigma}}}}}_1,{{{\mathbf{{\Sigma}}}}}_{21},{{{\mathbf{{\Sigma}}}}}_{22},{{{\mathbf{{\Sigma}}}}}_3 \sim {{{\mathrm{LJKCorr}}}}\left( 1 \right)$. We assumed that there was no correlation between the various compartments.

We used models that consider individual differences through a hierarchical structure for describing and predicting individual profiles.

**Fig. 5:**
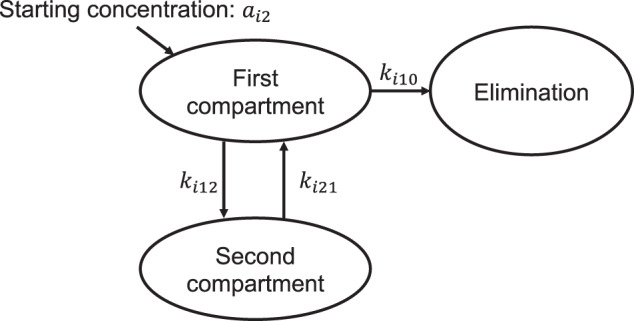
Diagram of the finally fitted two-compartment model. Each parameter is defined as follows: $k_{i21} = {\textstyle{{A_{i2}d_{i2} + C_{i2}b_{i2}} \over {A_{i2} + C_{i2}}}}$, $A_{i2} = \exp \left( {a_{i2}} \right)$, $C_{i2} = \exp \left( {c_{i2}} \right)$, $k_{i10} = {\textstyle{{b_{i2}d_{i2}} \over {k_{i21}}}}$, and $k_{i12} = b_{i2} + d_{i2} - k_{i21} - k_{i10}$.

The four candidate models were fitted using Stan in R, software version 4.1.2 (R Foundation for Statistical Computing, Vienna, Austria)^23^. We used a Hamiltonian Monte Carlo algorithm to generate samples from the posterior distributions of the parameters. We then evaluated the sampling convergence using trace plots and the Gelman–Rubin statistic, $\hat R$^24^, which was confirmed to be >1.01 for all parameters. The predictive performances of the models were compared using the LOOIC^25^. Fig. 5 depicts the best-fit model, that is, the two-compartment model. We estimated the posterior predictive distributions for the prediction of antibody titers with 95% prediction intervals for each time instant. We derived the time instants at which each participant’s lower limit of the 95% prediction interval falls below the 154 BAU/mL threshold for classification as a positive sample.

### Internal validation

We used grouped 10-fold cross-validation for prediction to assess the internal validity of the best-fit model described above. First, we divided the dataset into 10 equal parts and used nine to train and one to test. Thereafter, we randomly sampled one of the 3-, 8-, and 13-week measurements from each subject in the test data and used these as inputs to obtain 95% prediction intervals for $\hat Y_{it}$ at 26 weeks. We obtained prediction intervals by fitting a best-fit model using the nine parts of the training data. We then assessed whether these 95% prediction intervals included the actual values of *Y*_*it*_, root mean squared errors (RMSEs), and Pearson’s correlation coefficient between log *Y*_*it*_ and $\log \left\{ {{{{\mathrm{median}}}}\left( {\hat Y_{it}} \right)} \right\}$ at 26 weeks. We repeated this process, in which each of the ten parts participated in the test once. We defined the accuracy and RMSE at a time *t* as follows: accuracy = ((no. of patients whose 95% prediction interval included the actual value)/(no. patients)) × 100 [%], and ${{{\mathrm{RMSE}}}}_t = \sqrt {{\textstyle{1 \over n}}\mathop {\sum}\nolimits_{i = 1}^n {\left[ {\log Y_{it} - \log \left\{ {{{{\mathrm{median}}}}\left( {\hat Y_{it}} \right)} \right\}} \right]^2} }$.

### External validation

We assessed the external validity using three datasets (Mie National Hospital workers, *N* = 165; JSDT dialysis patients, *N* = 192; JSDT nondialysis elderly patients, *N* = 100), which were different from those used in the development of the best-fit model. We used different assay kits for different target populations and evaluated these to validate the generalizability of the best-fit model. Mie National Hospital is located in western Japan, where the SARS-CoV-2 infection rates were low. The participants from Mie National Hospital were healthy medical workers. Participants from JSDT were dialysis and nondialysis elderly patients; therefore, a different assay kit from that used for the Mie National Hospital participants was used.

For the Mie National Hospital data, we used measurements for each subject at 3.5 or 12 weeks as inputs to obtain 95% prediction intervals for $\hat Y_{it}$ at 24 weeks. For JSDT, we used the measurements for each subject at two weeks as inputs to obtain 95% prediction intervals for $\hat Y_{it}$ at 24 weeks. We performed external validation on three populations, that is, Mie Hospital workers, dialysis patients at JSDT, and nondialysis elderly patients at JSDT. We assessed whether the 95% prediction intervals included the actual values of *Y*_*it*_, RMSEs, and Pearson’s correlation coefficient at 24 weeks.

### Reporting summary

Further information on research design is available in the Nature Research Reporting Summary linked to this article.

## Supplementary information

Supplement Information

Video

REPORTING SUMMARY

## Data Availability

Derived data supporting the findings of this study (project name: COVID Vaccine Navi) are available on request from the corresponding author (Y.S.).
