# Supplementary material for: Estimating immunity with mathematical models for SARS-CoV-2 after COVID-19 vaccination
Source: NPJ Vaccines. 2023 Mar 6;8:33. doi: 10.1038/s41541-023-00626-w (PMC9988198; doi:10.1038/s41541-023-00626-w)

Supplementary information

Supplementary Table 1. Participant demographics of three datasets

Name of dataset	Keio University	Mie National Hospital	JSDT	
Area	Tokyo (capital city)	Mie (rural area, west Japan)	Tokyo (capital city)	
Affiliation	Healthcare workers	Healthcare workers	HD patients	Patients with underlying diseases (Not on HD)
Periods of 1st dose administration	05/03/2021 -12/03/2021	19/02/2021 - 05/03/2021	26/05/2021 - 31/08/2021	01/06/2021 - 14/08/2021
Reagents used for antibody measurement	Alinity SARS-CoV-2 IgG II reagents (Abbott; IL, USA)	enzyme-linked immunoassay-based kit (Denka Co. Ltd.; Tokyo, Japan)	VITROS Anti-SARS-CoV-2 IgG Chemiluminescent Immunoassay (Ortho Clinical Diagnostics; NJ, USA)	
The type and epitope of the measured antibody	IgG for RBD of spike protein	IgG for spike protein	IgG for spike protein	
Number	673	165	193	100
Median age, years (IQR)	45 (35 – 53)	41 (32-53)	68 (53-74)	67 (57-72)
Male participants (%)	196 (29.1)	40 (24.2)	123 (63.7)	61 (61.0)
Female participants (%)	477 (70.9)	125 (75.8)	70 (36.3)	39 (39.0)
History of COVID-19 (%)	10 (1.5)	0(0)	0 (0)	0 (0)

Immunosuppressant* use (%)	18 (2.7)	-†	0 (0)	0 (0)
Diabetes (%)	4 (0.6)	-†	92 (47.4)	17 (17.0)
Antigen test- or PCR-validated breakthrough infection during study period (%)	2 (0.3)**	0 (0)	0 (0)	0 (0)

Abbreviations; JSDT, Japanese Society for Dialysis Therapy; HD, hemodialysis; IQR, interquartile range

*Systemic use of steroids and other medications suppress cellular immunity.

†Data about immunosuppressant use and history of diabetes were not obtained from the healthcare workers of Mie National Hospital

**The data of participants whose antibody titer elevated between serial blood collection points were excluded from model building and validation.

Supplementary Table 2. Posterior distributions of the population level parameters for the final model

Parameter	Median	95% credible interval (CrI)	
μ_a	9.772	9.705	9.836
μ_b	0.256	0.246	0.266
μ_c	8.244	8.116	8.365
μ_d	0.058	0.054	0.062
σ_a	0.802	0.755	0.852
σ_b	0.031	0.025	0.037
σ_c	0.935	0.866	1.013
σ_d	0.019	0.017	0.021
σ_y	0.070	0.066	0.074
ρ_1	–0.195	–0.430	0.027
ρ_2	0.533	0.439	0.619

Supplementary Figure 1. Diagnostic results for the best fit model (trace plot).

Diagnostics of the best-fit model show that each parameter converges to a stable posterior distribution.

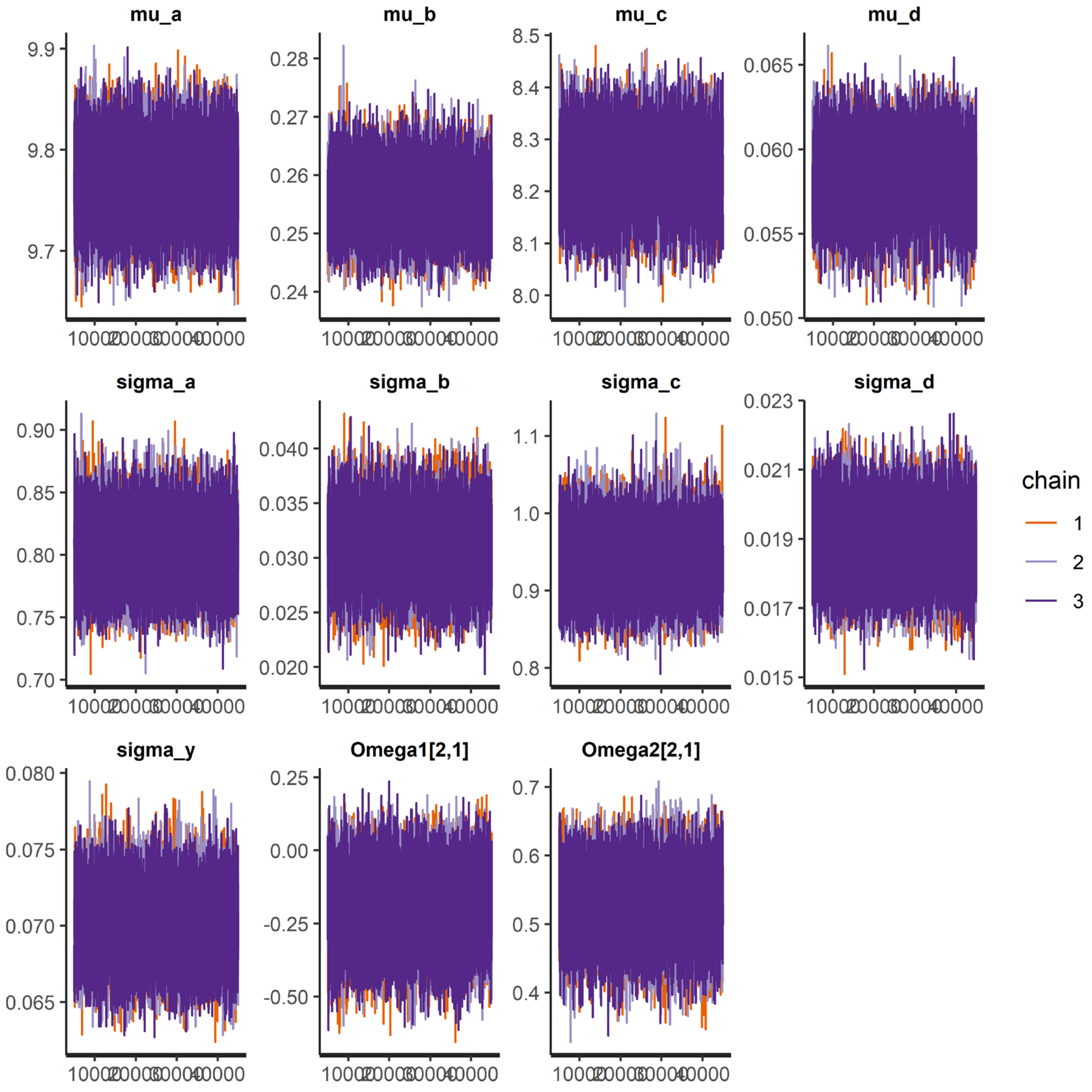

Supplementary Figure 2. Diagnostic results for the best fit model (density plot).

Diagnostics of the best-fit model show that each parameter converges to a stable posterior distribution.

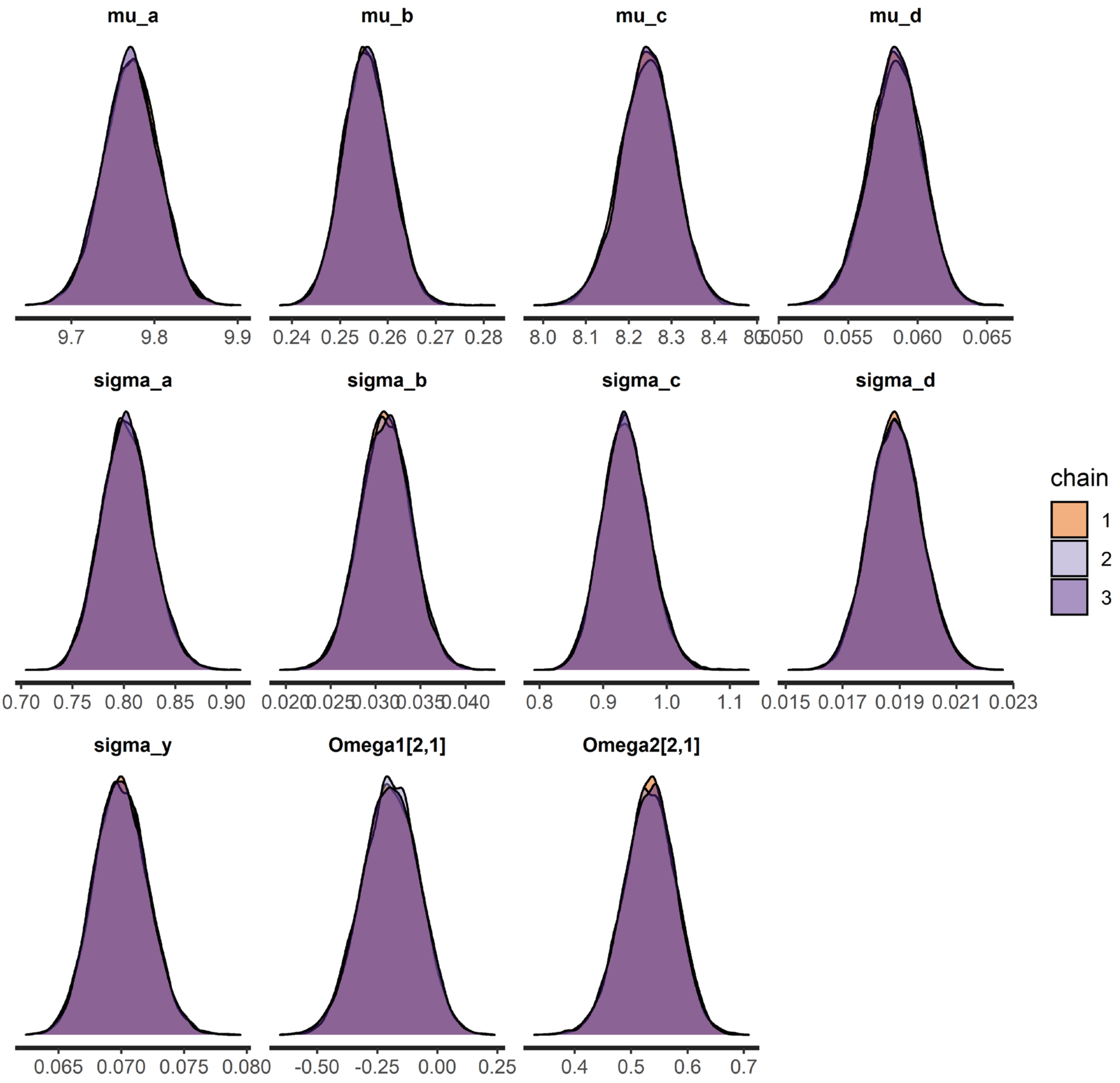

Supplementary Figure 3. Diagnostic results for the best fit model (auto correlation plot).

Diagnostics of the best-fit model show that each parameter converges to a stable posterior distribution.

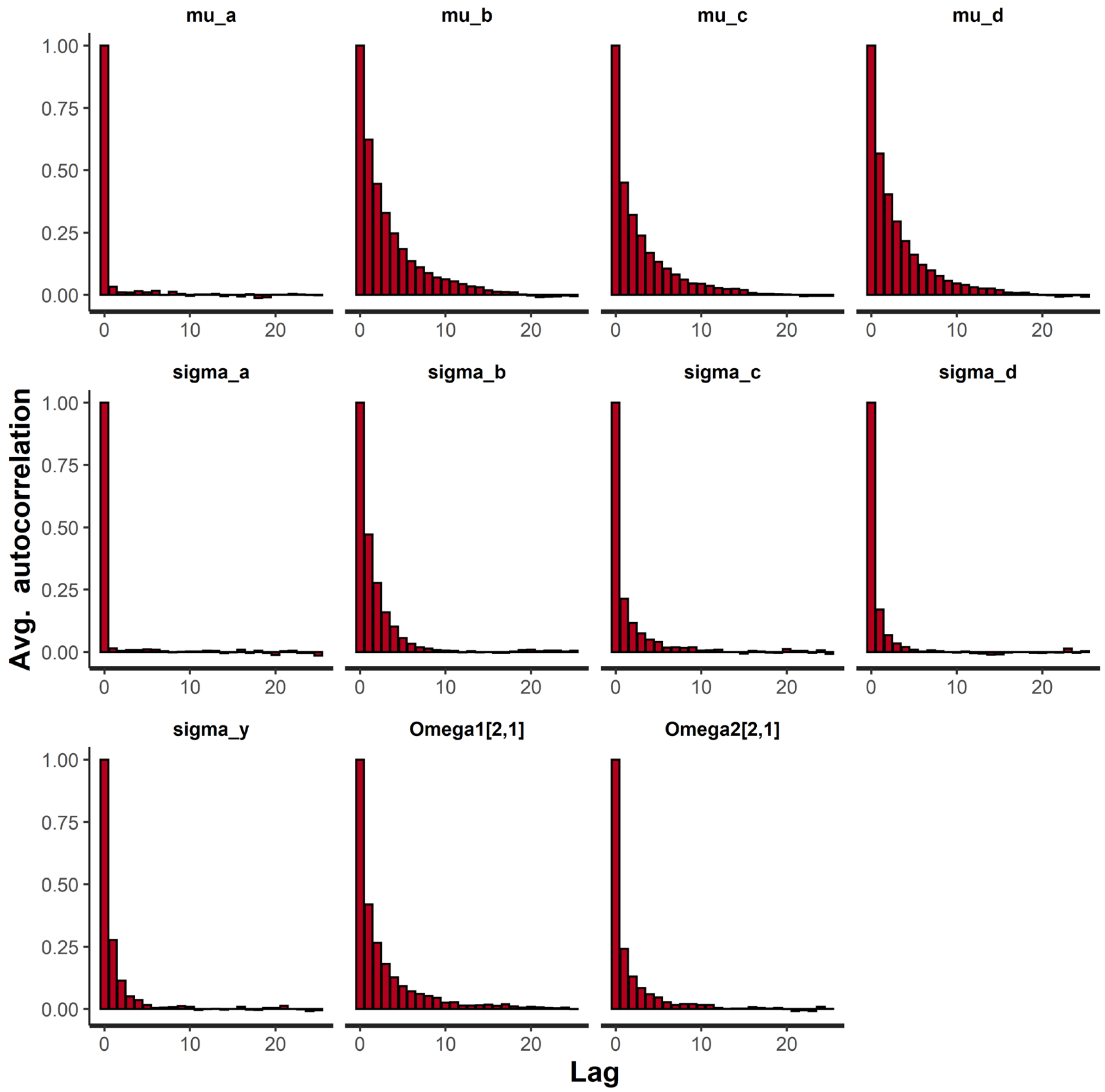

Supplementary Figure 4. Diagnostic results for the best fit model (effective sample size).

Diagnostics of the best-fit model show that the quality of the MCMC samples was sufficiently good.

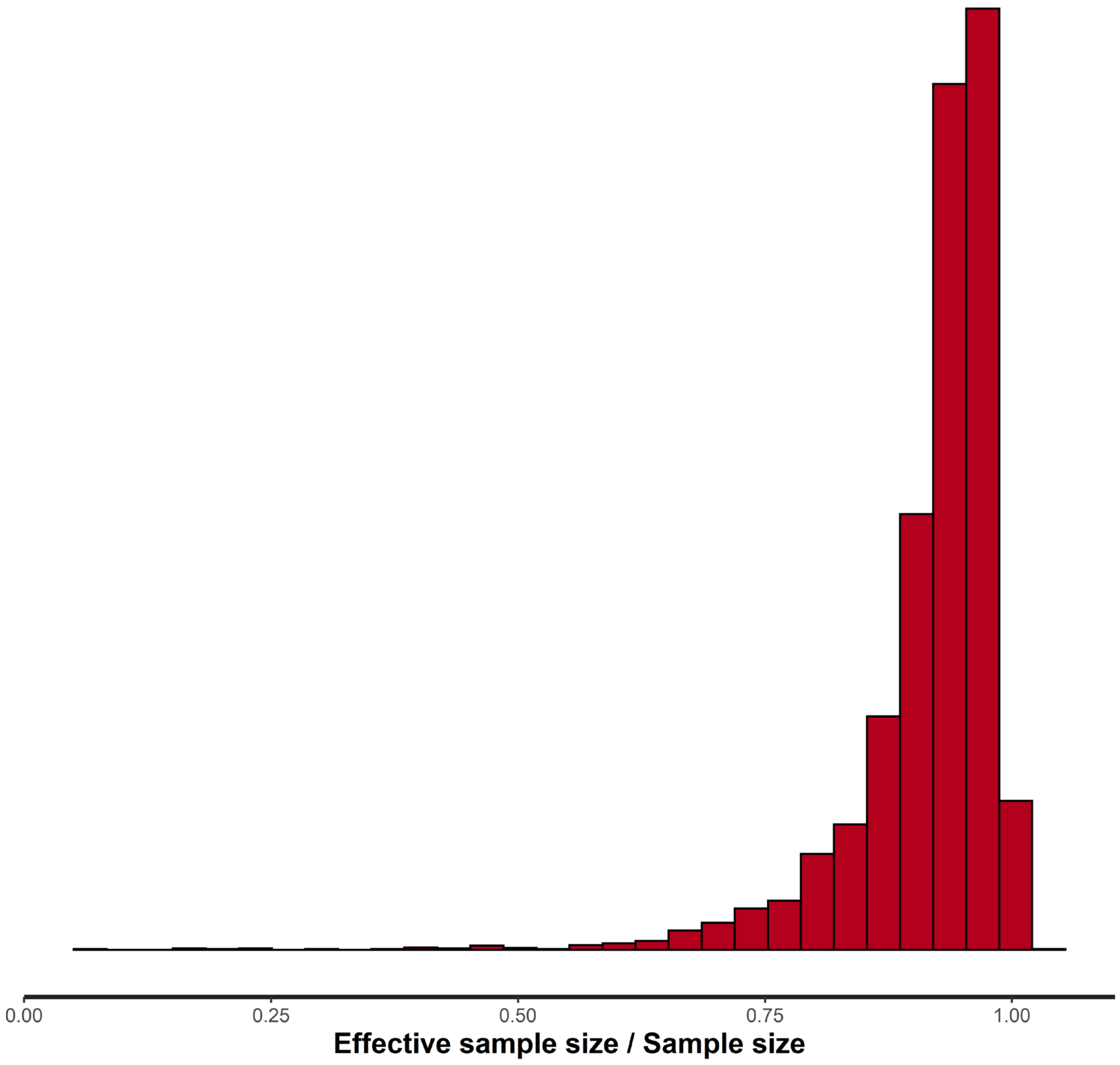

Supplementary Figure 5. Prediction results from Mie National Hospital data.

Prediction results from fitting the best-fit model to three selected participants from Mie National Hospital (IgG for spike protein was measured.). The area to the right of the vertical reference line displays the predicted results. Data and predicted results of antibody titers (antireceptor binding domain IgG) for a participant whose antibody titer increased to approximately 1,500 BAU/mL (a), 2,500 BAU/mL (b), and 3,500 BAU/mL (c) after two vaccination doses. Time instants are shown when the lower limit of the 95% prediction interval and median value fall below the 154 BAU/mL threshold, the horizontal reference line, for classification as a protective antibody titer. The vertical axis represents the logarithmic scale.

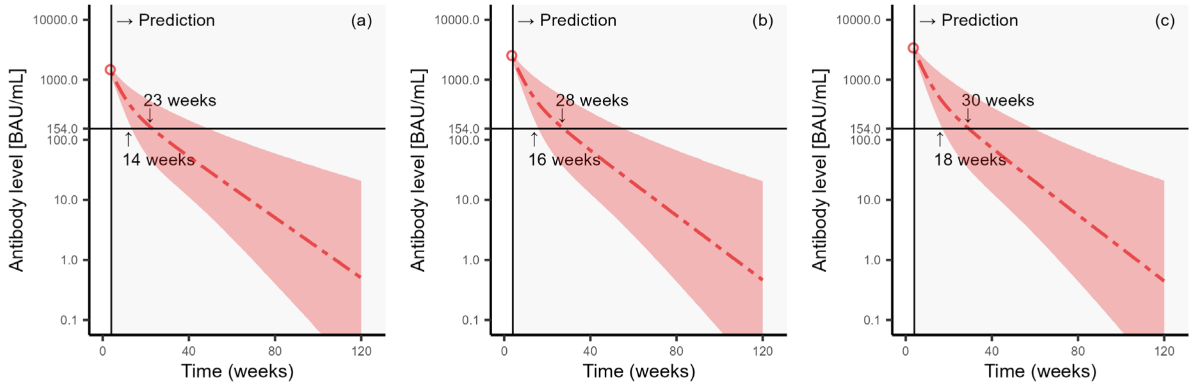

Supplementary Figure 6. Prediction results from the Japanese Society for Dialysis Therapy (JSDT) data: dialysis patients.

Prediction results from fitting the best-fit model to three selected hemodialysis patients from JSDT. The area to the right of the vertical reference line shows the predicted results. The area to the right of the vertical reference line displays the predicted results. Data and predicted results of antibody titers (antireceptor binding domain IgG) for a participant whose antibody titer increased to approximately 1,500 BAU/mL (a), 2,500 BAU/mL (b), and 3,500 BAU/mL (c) after two vaccination doses. Time instants are shown when the lower limit of the 95% prediction interval and median value fall below the 154 BAU/mL threshold, the horizontal reference line, for classification as a protective antibody titer. The vertical axis represents the logarithmic scale.

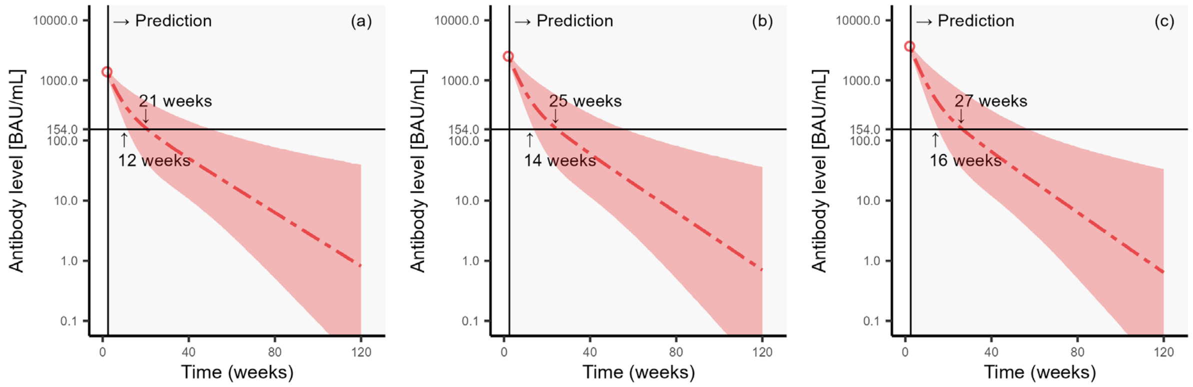

Supplementary Figure 7. Prediction results from the Japanese Society for Dialysis Therapy (JSDT) data: healthy elderly people.

Prediction results from fitting the best-fit model to three selected non-dialysis patients from JSDT. The area to the right of the vertical reference line shows the predicted results. The area to the right of the vertical reference line displays the predicted results. Data and predicted results of antibody titers (antireceptor binding domain IgG) for a participant whose antibody titer increased to approximately 1,500 BAU/mL (a), 2,500 BAU/mL (b), and 3,500 BAU/mL (c) after two vaccination doses. Time instants are shown when the lower limit of the 95% prediction interval and median value fall below the 154 BAU/mL threshold, the horizontal reference line, for classification as a protective antibody titer. The vertical axis represents the logarithmic scale.

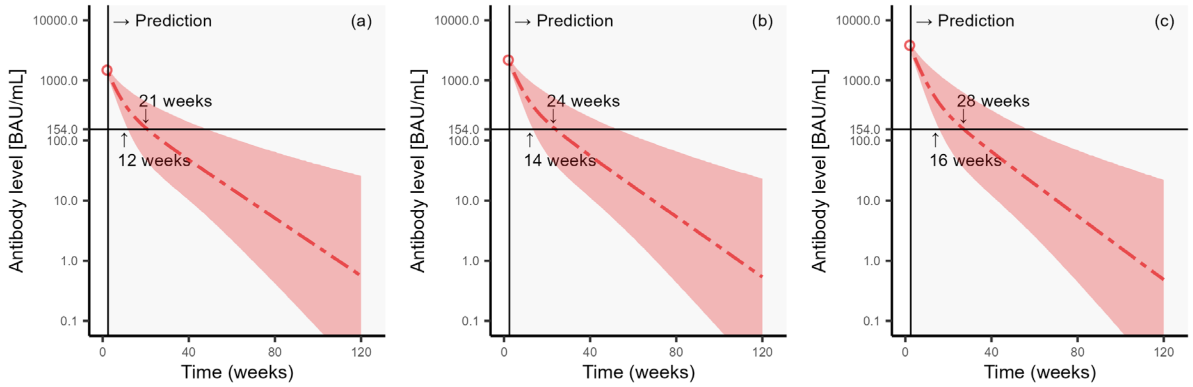

Supplement: Supplementary file 1 — Supplement Information [file 41541_2023_626_MOESM1_ESM.pdf]
