# Supplementary material for: Estimating immunity with mathematical models for SARS-CoV-2 after COVID-19 vaccination
Source: NPJ Vaccines. 2023 Mar 6;8:33. doi: 10.1038/s41541-023-00626-w (PMC9988198; doi:10.1038/s41541-023-00626-w)
Supplement: Supplementary file 3 — REPORTING SUMMARY [file 41541_2023_626_MOESM3_ESM.pdf]

nature portfolio

Corresponding author(s): Yasunori Sato
Last updated by author(s): Feb 7, 2023

Reporting Summary

Nature Portfolio wishes to improve the reproducibility of the work that we publish. This form provides structure for consistency and transparency in reporting. For further information on Nature Portfolio policies, see our Editorial Policies and the Editorial Policy Checklist.

Statistics

For all statistical analyses, confirm that the following items are present in the figure legend, table legend, main text, or Methods section.

n/a	Confirmed	
☐	☒	The exact sample size (*n*) for each experimental group/condition, given as a discrete number and unit of measurement
☐	☒	A statement on whether measurements were taken from distinct samples or whether the same sample was measured repeatedly
☒	☐	The statistical test(s) used AND whether they are one- or two-sided<br>*Only common tests should be described solely by name; describe more complex techniques in the Methods section.*
☐	☒	A description of all covariates tested
☒	☐	A description of any assumptions or corrections, such as tests of normality and adjustment for multiple comparisons
☐	☒	A full description of the statistical parameters including central tendency (e.g. means) or other basic estimates (e.g. regression coefficient) AND variation (e.g. standard deviation) or associated estimates of uncertainty (e.g. confidence intervals)
☒	☐	For null hypothesis testing, the test statistic (e.g. *F*, *t*, *r*) with confidence intervals, effect sizes, degrees of freedom and *P* value noted<br>*Give P values as exact values whenever suitable.*
☐	☒	For Bayesian analysis, information on the choice of priors and Markov chain Monte Carlo settings
☒	☐	For hierarchical and complex designs, identification of the appropriate level for tests and full reporting of outcomes
☒	☐	Estimates of effect sizes (e.g. Cohen's *d*, Pearson's *r*), indicating how they were calculated

Our web collection on statistics for biologists contains articles on many of the points above.

Software and code

Policy information about availability of computer code

Data collection	No code was used to collect the data analyzed in this study.
Data analysis	The models were fitted by using Stan in R software version 4.1.2 (R Foundation for Statistical Computing; Vienna, Austria).

For manuscripts utilizing custom algorithms or software that are central to the research but not yet described in published literature, software must be made available to editors and reviewers. We strongly encourage code deposition in a community repository (e.g. GitHub). See the Nature Portfolio guidelines for submitting code & software for further information.

Data

Policy information about availability of data

All manuscripts must include a data availability statement. This statement should provide the following information, where applicable:

- Accession codes, unique identifiers, or web links for publicly available datasets
- A description of any restrictions on data availability
- For clinical datasets or third party data, please ensure that the statement adheres to our policy

The datasets generated during and/or analysed during the current study are available from the corresponding author on reasonable request.

Human research participants

Policy information about studies involving human research participants and Sex and Gender in Research.

Reporting on sex and gender	Sex of participants was determined based on assigned. Sex-based analyses have been performed.
Population characteristics	Population characteristics for the dataset are given in Supplementary Table S1 .
Recruitment	Individuals were recruited for research at Keio University Hospital, National Health Organization Mie National Hospital, and Infection Control Committee of the Japanese Society for Dialysis Therapy (JSDT) after (prospective) they received two doses of BNT162b2 vaccine.
Ethics oversight	Ethical Committee of Keio University School of Medicine (approval No. 20210301).<br>Ethics Committee of the National Hospital Organization Mie National Hospital (approval NO. 2021-141).<br>Ethics Committee of the JSDT (approval numbers 1-10).

Note that full information on the approval of the study protocol must also be provided in the manuscript.

Field-specific reporting

Please select the one below that is the best fit for your research. If you are not sure, read the appropriate sections before making your selection.

☒ Life sciences ☐ Behavioural & social sciences ☐ Ecological, evolutionary & environmental sciences

For a reference copy of the document with all sections, see nature.com/documents/nr-reporting-summary-flat.pdf

Life sciences study design

All studies must disclose on these points even when the disclosure is negative.

Sample size	No sample-size calculations were performed for this study.
Data exclusions	Cases for which data were available only at week zero were excluded from analyses.
Replication	Replication was not performed in this study. However, 10-fold cross-validation and external validation were used to avoid over-fitting of the compartmental model.
Randomization	Randomization is not relevant to this study.
Blinding	Blinding was not relevant to this study.

Reporting for specific materials, systems and methods

We require information from authors about some types of materials, experimental systems and methods used in many studies. Here, indicate whether each material, system or method listed is relevant to your study. If you are not sure if a list item applies to your research, read the appropriate section before selecting a response.

Materials & experimental systems

n/a	Involved in the study	
☐	☒	Antibodies
☒	☐	Eukaryotic cell lines
☒	☐	Palaeontology and archaeology
☒	☐	Animals and other organisms
☒	☐	Clinical data
☒	☐	Dual use research of concern

Methods

n/a	Involved in the study	
☒	☐	ChIP-seq
☒	☐	Flow cytometry
☒	☐	MRI-based neuroimaging

Antibodies

Antibodies used	[Keio University Hospital]<br>The IgG antibody titers for SARS-CoV-2 spike proteins were measured using Alinity SARS-CoV-2 IgG II reagents and Alinity analyzer (Abbott; IL, USA).

[National Health Organization Mie National Hospital]
The IgG antibody titer for SARS-CoV-2 spike proteins of consecutively obtained serum samples were measured by an enzyme-linked immunoassay-based kit (Denka Co. Ltd.; Tokyo, Japan) which was certified by WHO international standard serum samples.

[JSDT]
Antibody titers obtained from JSDT were measured using the Ortho-Clinical Diagnostics VITROS® Anti-SARS-CoV-2 IgG Chemiluminescent Immunoassay, which was also certified by WHO international standard serum samples.

Validation

All reagents used in the study were validated by manufacturers using WHO International Standard for anti-SARS-CoV-2.
